# Effect of blood flow-restrictive resistance training on metabolic disorder and body composition in older adults with type 2 diabetes: a randomized controlled study

**DOI:** 10.3389/fendo.2024.1409267

**Published:** 2024-06-06

**Authors:** Xiaojun Ma, Yuxin Ai, Fulian Lei, Xuan Tang, Qingmei Li, Yixin Huang, Yating Zhan, Quan Mao, Liduo Wang, Fenfang Lei, Qinyu Yi, Fan Yang, Xiaogang Yin, Binghua He, Lei Zhou, Sijie Ruan

**Affiliations:** ^1^ School of Nursing, Shaoyang University, Shaoyang, Hunan, China; ^2^ Department of Endocrinology, The Second Affiliated Hospital of Shaoyang University, Shaoyang, Hunan, China; ^3^ Department of Anesthesiology, Central Hospital of Shaoyang, Shaoyang, Hunan, China

**Keywords:** blood flow-restrictive resistance exercise, type 2 diabetes, metabolic disorders, body composition, older adults

## Abstract

**Introduction:**

To explore whether blood flow-restrictive resistance exercise (BFRE) can be used as an alternative strategy to moderate-intensity resistance training (RT) to improve metabolic disorder and body composition in older adults with type 2 diabetes (T2DM).

**Methods:**

This is a single-blind, randomized, controlled trial. Ninety-eight older adults with T2DM were randomly divided into three groups: BFRE group (n = 34), RT group (n = 31) and control group (n = 33). Two exercise groups received supervised collective training for a period of six months, each lasting 50 min, three times a week. The primary outcomes included fasting plasma glucose (FPG), Glycosylated hemoglobin (HbA1c), blood lipids, blood pressure, and body composition. The secondary outcome was muscle performance.

**Results:**

After six months of intervention, the FPG, HbA1c, blood lipids, diastolic blood pressure, body composition, and muscle performance of the two exercise groups were significantly improved relative to the control group and baseline measurements (P < 0.05). There was no significant increase in lean mass between the two exercise groups compared to the control group and baseline (p > 0.05). There was no significant decrease in systolic blood pressure between the two exercise groups compared to the control group (p > 0.05), but it was significantly lower than their baseline (P < 0.05). There was no significant difference in all indicators between the two exercise groups at the baseline, third and sixth months of intervention (p > 0.05).

**Discussion:**

BFRE can safely and effectively improve the metabolic disorder and body composition of older adults with T2DM. For elderly exercise beginners, BFRE can be used as an alternative strategy to moderate-intensity resistance training.

**Clinical trial registration:**

https://www.chictr.org.cn/showproj.html?proj=178886, identifier ChiCTR2300074357.

## 1 Introduction

The number of older adults with diabetes is increasing with the aging of society (1, 2). The aging process leads to changes in human body composition. One of the characteristics is the continuous and inevitable decline of muscle mass (3). The loss of muscle mass reduces the quality of available insulin-responsive target tissues, thus promoting insulin resistance and leading to the occurrence of diabetes (4). Increasing skeletal muscle mass helps regulate glucose use, lipid oxidation, and resting metabolic rate.

On the basis of a healthy diet, ensuring sufficient exercise is the most effective way to enhance skeletal muscle. Therefore, many guidelines (2) recommend that diabetic patients should perform a total of 150 min of moderate- to high-intensity exercise at least three days a week, including two-three days of resistance training (RT). Classic resistance training [60–80% of an individual’s one-repetition maximum (1-RM)] has proven to be effective in improving the mass and strength of skeletal muscle and controlling blood glucose (5). Unfortunately, due to a sedentary lifestyle, fatigue, pain, cardiovascular and cerebrovascular diseases, as well as concerns about safety and lack of professional guidance, so that elderly diabetes patients find it difficult to tolerate and adhere to high-intensity exercise training (6). Older people are more inclined to choose exercise methods with high safety factors and low intensity. However, low-intensity exercise not only requires athletes to achieve exhaustion, but also consumes more time and has significantly poorer effects compared to high-intensity training (7). Therefore, there is an urgent need to find a relatively low-intensity exercise that is easy to accomplish, safe, and effective as an alternative strategy that can match the beneficial effects of moderate- to high-intensity exercise.

A promising exercise method to achieve this goal is blood flow-restrictive resistance exercise (BFRE), which is a new exercise method that combines distal ischemic preconditioning and low-intensity resistance training (8). The main difference from classical resistance exercise is reduced blood flow to the moving limbs (9). Numerous studies (10, 11) have shown that the skeletal muscle hypertrophy and strength enhancement effects observed in BFRE using only 20–30% 1-RM are comparable to those observed in moderate- to high-intensity resistance training (≥ 70% 1-RM) (12). Wang et al. (13) concluded that after six weeks of training with BFRE in healthy older adults, BFRE was more effective in stimulating skeletal muscle growth and improving muscle function in the elderly compared to the non-exercise control group. Therefore, they advocated the use of BFRE as a strategy to prevent age-related deterioration of skeletal muscle mass and function. Christiansen et al. (14) conducted a six-week study of exercise training in healthy men, in which one leg was trained with BFRE and the other leg without BFRE. The results indicated that the skeletal muscles of the leg trained with BFRE significantly increased glucose intake. This may be due to BFRE promoting a significant increase in muscle antioxidant function, GLUT4 abundance, and/or nitric oxide availability.

Compared to people of the same age and with normal blood glucose, older adults with T2DM experienced accelerated loss of muscle mass and strength (15), and had a higher risk of hypoglycemia, thrombosis, and cardiovascular and cerebrovascular diseases (16). The existing BFRE research is mainly aimed at people who are non-diabetic. Whether BFRE can safely and effectively control abnormal glucose and dyslipidemia by improving the body composition of older adults with T2DM remains unknown.

To address this problem, the effect of low-intensity BFRE on the abnormal metabolism of glucose and lipids was evaluated, along with improvements on human body composition in older adults with T2DM during a six-month, supervised exercise intervention. The results from BFRE and moderate-intensity resistance exercise were compared to explore whether BFRE can become an alternative strategy. The aim of this study is to provide more extensive exercise choices for older adults with T2DM, improve their life years, and provide a reference for exercise-based prevention and treatment of diabetes.

## 2 Materials and methods

### 2.1 Study design and participants

Participants were recruited from March to May 2023 at the three community health service centers in Daxiang District, Shaoyang City, Hunan Province, through medical staff referrals, physical examinations, lectures, broadcasts, and advertisements, etc. in the streets under their jurisdiction. The main inclusion criteria to meet eligibility for this study were: 60–80 years old, diagnosed with T2DM according to WHO diagnostic criteria (17), sedentary (exercise< 1 hour per week), not using an insulin pump, HbA1c levels of 6.5–11.0%, stable weight (weight loss or increase of no more than 2 kg), stable medication within three months before registration, and those who signed informed consent and volunteered to participate. The exclusion criteria mainly included guidelines (2, 18–20) and research (16) that indicated that individuals with contraindications to exercise, cognitive impairment, use of drugs that affect body composition, or inability to complete predetermined exercise programs were not suitable for inclusion in this study. Before randomization, participants were guided by exercise rehabilitation therapists to perform an incremental load test using a power vehicle for a duration of 20 minutes. The exercise test comprehensively evaluated cardiopulmonary reserve function, reserve oxygen uptake, and metabolic equivalents to assess the safety of the participants during exercise. Personal baseline data was recorded to develop personalized exercise programs (21). After obtaining medical approval from a physical therapist and endocrinologist, all participants were assigned to different exercise groups and completed corresponding exercise programs as required, with a total intervention period of six months (24 weeks).

### 2.2 Ethical considerations

This study protocol was reviewed and approved for implementation by the Medical Ethics Committee of Shaoyang University in accordance with the International Ethical Guidelines for Biomedical Research Involving Human Subjects and the Declaration of Helsinki. In addition, this study is registered on the Chinese Clinical Trials Registry (Registration No.: ChiCTR2300074357). All participants voluntarily participated in the study and signed informed consent forms. Participants had the right to withdraw from the study at any time for any reason without any consequences for further treatment, and the data set is kept confidential.

### 2.3 Randomization and blinding

This study was a single-blind, randomized, controlled trial, in which an independent chief researcher assigned unique codes to anonymize participant information according to inclusion order. Then, participants were randomly assigned to three parallel groups based on a computer-generated numerical sequence: the control group, the resistance training group (RT), and the blood flow restrictive resistance exercise group (BFRE). The researchers responsible for each group intervention and evaluation were independent of each other, and the evaluators were blinded to each participant’s group assignment.

### 2.4 Sample size

The necessary sample size was estimated as follows: $\text{n}=\frac{\Psi 2(\sum_{\text{i}=1}^{\text{k}}{\text{S}}_{i}^{2}/\text{k})}{\sum_{\text{i}=1}^{\text{k}}({\left(\chi_{\text{i}}-\chi \right)}^{2}/(\text{k}-1)}$ . In our pilot study, the mean and standard deviation (mean ± SD) of fasting plasma glucose (FPG) for the three groups were 8.64 ± 1.25 for the control group, 7.51 ± 1.31 for the RT group, and 8.01 ± 1.18 for the BFRE group. One – way ANOVA of α = 0.05 (bilateral), β = 0.10, Ψ=2.55 were taken. To test whether there are significant differences among the three groups in the final data analysis, at least 31 participants were needed in each group. Therefore, the total sample size was calculated as 93. Based on a dropout rate of 20%, this required at least 38 participants per group, or a total of 114 participants.

### 2.5 Intervention measures

#### 2.5.1 Diabetes education

Before the exercise intervention, all participants were invited to join different WeChat groups according to their grouping. We sent notifications and diabetes education materials to them through WeChat, and arranged for them to participate in diabetes health education classes at different times and stages, including diabetes healthy diet, exercise regularly, medication, blood glucose monitoring and regular follow-up appointments. The teaching format was online and offline blended teaching over a total of 13 hours (4 hours before the intervention, 1.5 hours per month of the intervention). All courses were administered by a diabetes-specialist nurse with 12 years of work experience. Before the end of each course, all participants were scheduled to take a test to ensure that they mastered the relevant knowledge.

#### 2.5.2 Control group

During the study, participants were recommended to change their lifestyle according to our diabetes education content. However, supervised exercise interventions and detailed resistance training programs were not provided; participants could adhere to the advice to exercise and adjust their diet on their own or maintain their original lifestyle habits. A researcher recorded the daily exercise habits of participants through a fitness tracker or WeChat exercise mini-program, mobile health management software. Participants in the control group were invited to participate in physical examinations and face-to-face interviews before the intervention and at the third and sixth months of follow up appointments.

#### 2.5.3 exercise intervention

The exercise intervention was conducted at the sports fields near the three community health service centers. The baseline values of the participants were measured before the exercise programs began. The first two weeks of the formal exercise intervention were for adaptation training to help participants develop exercise programs based on their individual baseline levels and familiarize themselves with the training process. Two professionally trained researchers supervised and led participants in training at each sports field to ensure standardized movements, achieve predetermined intensity, and ensure safety. After conducting a motivation survey, the exercise time was set between 7:30–8:30 in the evening. Thirty minutes before each exercise, the researchers asked the participants about their diet and physical condition, and measured their blood glucose, blood pressure, and heart rate (HR). Both exercise groups warmed up for ten minutes before the main training session, which lasted for 30 minutes. A heart rate monitoring bracelet was used to dynamically monitor the HR of participants during exercise to maintain a moderate-intensity of exercise (40–59% HR reserve). Afterwards, they stretched and relaxed for ten minutes, for a total of 50 minutes. The exercise sessions were provided three times a week, with 24–48 hours’ interval between each session, over a total of six months (24 weeks). If participants participated a total of 15 times a month, their attendance rate was considered to be 100%; if their attendance was ≥ 10 times per month (≥ 70% attendance rate), they were considered to have met the standard (22); if their attendance was less than seven times per month (< 50% attendance rate) (5), they were excluded if their attendance did not improve after encouragement and communication.

##### 2.5.3.1 RT group

Resistance training was performed using small equipment such as barbells, dumbbells, elastic bands/ropes, and kettle bells because participants were expected to be able to learn to use small equipment at home after the study was over. Upper body exercises (shoulder press and pull down, elbow extension and flexion), hip and leg exercises (leg press, extension and flexion), and core muscle group exercises (flat-ground support, glute bridge, push-ups, sit-ups) were mainly selected for training. The initial intensity of the strength training program was low (40%–50% 1-RM) to reduce muscle soreness, avoid Valsalva movements and tendon injuries, and ensure proper weight lifting form. If the participant could fully adapt, that was, repeat 10–15 times/set with the same resistance, complete 2–4 sets, and self-rate the degree of fatigue during and after exercise to achieve 12–13 points of the Borg ratings of perceived exertion (RPE) scale, resistance can be gradually increased 5%–10% 1-RM until they can complete the moderate-intensity resistance training at a personal 60%-70% 1-RM, repeating 10–15 times/set, 60 seconds interval between sets (19, 23). **Table 1** shows the detailed resistance training scheme.

**Table 1:** Resistance training scheme.

Weeks	Intensity (% 1-RM)	Repetitions	Perceived Exertion (Rating on 6–20 RPE Scale)	Interval between sets	Frequency (times/ week)	Sets
1 (Pre-intervention)	40–50	8–12	9–11	1 minute	3	2–4
2 (Pre-intervention)	45–55	10–15	9–12			
1–2 (Intervention)	55–60	8–12	12–13			
3–4	55–60	10–15	12–13			
5–8	60–65	8–12	12–13			
9–12	60–65	10–15	12–14			
13–16	60–70	8–12	12–14			
17–24	60–70	10–15	12–14			

RPE, ratings of perceived exertion; 1-RM, one-repetition maximum.

##### 2.5.3.2 BFRE group

The KAATSU Air Band (Product type: C3, USA) was tied to the most proximal end of the subject’s upper or lower limb, with a tightness that could accommodate one finger. The KAATSU host was used to control the bandage to gradually apply pressure to the limb, while the laser Doppler flowmeter quantitative analyzer (SONIMAGE, product type: HS1, China) was used to test the minimum pressure required for the subject’s limb to be blocked by arterial blood flow. The pressure displayed on the KAATSU host at this time was referred to as the total limb occlusion pressure (LOP). During each training session, the cuffs were tied to the most proximal ends of the subject’s upper or lower limbs based on the main muscle groups trained, and blood flow was limited to 50% LOP of the individual’s limbs (24). At the same time, resistance training intensity was 20–30% 1-RM. The training equipment and movements were the same as those in the ST group. The volume followed a scheme from 30 repetitions in the first set and 15 repetitions in set three. The rest periods between the sets were 30 s with the cuffs remaining inflated during rest. The blood flow restriction lasted for a total of 6.5 minutes. Then, the cuffs were completely relaxed to allow blood reperfusion, during which time two sets of core muscle group trainings were continued. There was a rest period of 30 s between sets. The total time for blood flow restriction + reperfusion training was ten minutes for each round, with three rounds in total for a total of 30 minutes. It is important to note that the applied cuff pressure should not cause pain or any obvious discomfort in the subject during training, and can be adjusted to 40–50% LOP based on the comfort of the subject (25). The goal in this study was to use pressure sufficient to induce muscle adaptation while minimizing discomfort to avoid interruptions in exercise due to subcutaneous bleeding, thrombosis, limb soreness, and other problems in the subject. Information on this regimen is shown in **Table 2**.

**Table 2:** blood flow-restrictive resistance exercise training scheme.

Action	Time	Training location	Main movement
Inflation and pressurization	1 minute		Intensity: 20%–30% 1-RM. 10 minutes × 3 = 30 minutes
Repetitions: 1^st^ set: 30 → 2^nd^ set: 15→ 3^rd^ set: 15 → 4^th^ set: 15	A total restriction time = 6.5 minutes	Training on both upper or lower limbs with 40–50% LOP	
Interval between sets: 30 seconds			
Complete deflation and rest	30 seconds		
Continue resistance training without blood flow restriction	2 sets + 30 seconds rest × 2 = 2 minutes	Core muscles	

LOP, limb occlusion pressure; 1-RM, one-repetition maximum.

### 2.6 Outcome measures

The measurements were conducted at three time points: baseline (0 month), and in the third and sixth months of the intervention. The non-blood-test results were available at the end of the test, and the blood test results were reported the day after the blood collection. Researchers entered the three groups’ measurement data into Excel spreadsheets separately and conducted double checks to ensure the accuracy of the data. The data entry personnel and data analysts were independent of each other. Identity information of the subjects was replaced with corresponding numbers, and all qualified data were scanned and encrypted for storage. The original report forms were distributed to the subjects themselves, and no one was allowed to disclose patient privacy information.

#### 2.6.1 Baseline data

Before intervention, demographic and clinical characteristics of the subjects were investigated with structured questionnaires.

#### 2.6.2 Primary outcomes

##### 2.6.2.1 Measurement of FPG, HbA1c, blood lipids, and blood pressure

At three time points during the measurement, three groups of participants went to designated community health service centers for physical examination between 7:00 and 9:00 am. Venous blood samples were collected from the subjects after fasting for more than eight hours, then frozen at -80 °C. After centrifugation at 3000 rpm for 15 minutes, the serum was separated and analyzed. (1) HbA1c levels were measured using ion-exchange resin high-performance liquid chromatography by a Variant II HbA1c instrument (Bio-Rad, product type: 270–2001, USA). (2) A fully automatic biochemical analyzer instrument (Hitachi, product type: 7600, Japan) was used to measure levels of fasting plasma glucose (FPG), total cholesterol (TC), triglyceride (TG), low density lipoprotein cholesterol (LDL-C), and high density lipoprotein cholesterol (HDL-C). (3) After the subject rested for 15 minutes, an electronic blood pressure monitor (OmRon, product type: U730, China) was used to measure the blood pressure (BP), included the systolic blood pressure (SBP) and diastolic blood pressure (DBP). A medical professional instructed the subjects to straighten their arms and tie cuffs two centimeters above their elbow sockets. The tightness of the cuffs can accommodate one finger. The subject was seated with the brachial artery measurement point, blood pressure monitor, and heart position at the same level. They were kept quiet until the end of the blood pressure measurement, accurate to 1 mmHg.

##### 2.6.2.2 Body composition

A direct, segmented, multi-frequencies bioelectrical impedance analyzer (DSM-BIA) (InBody, product type: 770, Korea) was used to estimate body composition. The instrument was equipped with eight electrode touch points on both sides of the thumb, palm, sole, and heel. Five segments of the limbs and trunk were subjected to multi-frequency current through six different impedance frequencies (1, 5, 50, 250, 500, and 1000 kHz) and three different reactance frequencies (5, 50, and 250 kHz) to estimate body composition. Before the test, the subjects were required to fast and empty their urine, not exercise, or drink excessively for 30 minutes. They were asked to remove their metal jewelry, shoes, and socks; clean their hands and feet with a cloth; stand barefoot in the center of the instrument chassis; and slightly separate their legs. The forefoot and heel were placed on the front and rear foot electrodes on the instrument chassis, respectively. The weight was automatically measured to an accuracy of 0.01 kg. After entering the correct height of the subject into the system, the subject was prompted to place their thumbs and palms on the handle electrodes, stand straight, stretch their arms away from both sides of their body, remain quiet, and maintain their posture until the end of the test. Upon completion of the test, a test report was available for the researcher to enter the subject’s skeletal muscle mass (SMM), appendicular skeletal muscle mass (ASM), fat mass (FM), lean mass (LM), weight, and body mass index (BMI) into an Excel table and calculate the following indicators: fat mass index (FMI) = FM/Height^2^ (kg/m^2^), skeletal muscle mass index (SMI) = SMM/Height^2^ (kg/m^2^), appendicular skeletal muscle mass index (ASMI) = ASM/Height^2^ (kg/m^2^). Waist circumference and height were measured with a tape measure, accurate to 0.1 cm. Waist-to-height ratio (WHtR) was calculated based on waist circumference (cm)/height (cm); a WHtR > 0.5 indicates abdominal obesity. Obesity was evaluated based on BMI and divided into normal (18–23.9 kg/m^2^), overweight (24–27.9 kg/m^2^), and obese (≥ 28 kg/m^2^) (2).

#### 2.6.3 Secondary outcome

##### 2.6.3.1 Muscle performance

(1) Grip strength test: An electronic grip strength meter (CAMRY, product type: EH101, China) was used to estimate grip strength. During the test, the patient was required to stand straight, fully extend their arms, and squeeze the handle of the dynamometer with maximum strength for three seconds. The measurement was taken twice with both hands alternately. The maximum grip strength value was taken, accurate to 0.1 kg. (2) Five times-sit-to-stand test: The subject was instructed to always keep their arms crossed in front of their chest, stand up as quickly as possible from a 40 cm-high chair, and sit down again. The action was repeated five times and the time used was recorded, accurate to 0.01 seconds. (3) The 6-m walking speed test: The patient was instructed to walk straight at the fastest speed for a distance of 6 m, twice in total. The shortest time of the two records was taken, then the walking speed of the examinee was calculated, accurate to 0.01 m/s.

#### 2.6.4 Quality control

All exercise interventions were carried out under the supervision of the researchers. During this period, if the researcher discovered events such as hypoglycemia, high blood pressure, illness, injury, etc., the participant would be required to immediately suspend or terminate the experiment, and given treatment measures within their ability. Afterwards, the treatment process should have been supplemented and recorded. The researchers needed to truthfully report the handling of adverse events to the chief researcher to arrange subsequent treatment and necessary compensation. If it was not suitable to go out for training in extreme weather, online live streaming, video playback, and other methods to guide participants to complete training at home was provided. All other training was conducted in a collective form. To reduce the dropout rate, the research group also took measures, such as regularly holding diabetes-control themed activities, providing free physical examinations and medical consultations, and issuing incentive gifts and bonuses.

### 2.7 Statistical analysis

Statistical analysis was performed using SPSS 23.0 (SPSS Inc., Chicago, IL, USA). For the comparison of baseline demographic characteristics between groups, the analysis of variance (ANOVA) or Kruskal-Wallis test was used for quantitative data, and the chi-square test was used for count data. The independent sample t-test was used to compare the attendance rates of the two control groups. The two-factor repeated measures ANOVA was used to analyze the changes in each dependent variable over time (from baseline to six months), and to analyze the interaction between time and population. In the case of non-compliance with the Mauchly’s test of sphericity hypothesis, the results were analyzed using Greenhouse-Geisser correction. The Bonferroni correction was used for *post-hoc* multiple comparisons. The mean difference [95% Confidence Interval (CI)] within the group from post-measurement to baseline was represented by the mean difference. The effect sizes of repeated measurement ANOVA was expressed by partial eta-squared (η^2^p, small ≥ 0.01; medium, ≥ 0.06; large ≥ 0.14). The effect sizes of mean differences between groups were expressed by Cohen’s d (d; small, ≥ 0.2; medium, ≥ 0.5; large ≥ 0.8). The level of statistical significance was defined as 0.05.

## 3 Results

### 3.1 Participant demographics and clinical characteristics

**Figure 1:**
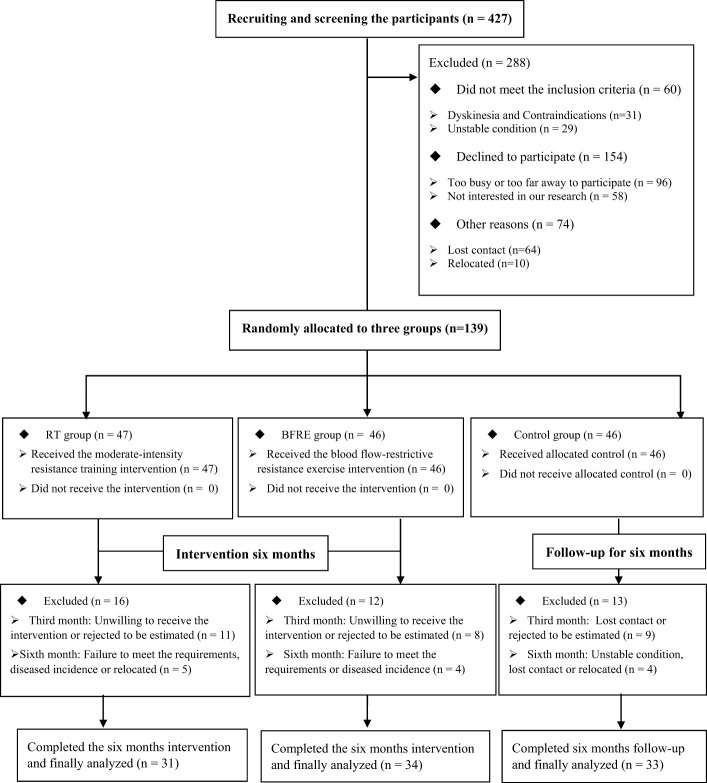
Flow chart of participant enrollment, allocation, and analysis.

As shown in **Figure 1**, in June 2023, 139 older adults with T2DM who received a qualification assessment were selected to meet the inclusion criteria. After six months of intervention (June 2023-December 2023), 41 participants were excluded due to non-compliance, disease, relocation, and other reasons. Therefore, a total of 98 participants completed this study, including 33 in the control group (dropout rate of 28.26%), 31 in the RT group (dropout rate of 34.04%), and 34 in the BFRE group (dropout rate of 26.09%). Their average attendance rate was ≥ 70%, with 82.37 ± 3.55% in the RT group and 81.74 ± 2.59% in the BFRE group. There was not significant difference in attendance rates between the two exercise groups (t = 0.818, p = 0.416, df = 63).

### 3.2 Baseline data

As shown in **Table 3**, the baseline data for each dependent variable among the three groups were consistent (p > 0.05). The average age of the participants was 66.19 ± 4.75 years old, with 39 males and 59 females, and an average height of 161.43 ± 8.80 cm. Among all participants, 91 (92.9%) had abdominal obesity (WHtR > 0.5), 88 (89.8%) were overweight/obese (BMI ≥ 24 kg/m^2^), 69 (70.41%) used antihypertensive drugs, and 32 (32.7%) used medicine to regulate dyslipidemia. All participants had diabetes for more than two years, used hypoglycemic drugs to control blood glucose, and used them steadily for at least three months. During the intervention period, no patients changed the way they used hypoglycemic drugs, but some patients had to adjust their drug dosage due to changes in their condition. Four patients in the control group increased their dosage, while two patients in the RT group and two patients in the BFRE group needed to lower their dosage. There was no significant difference in drug adjustment among the three groups of participants (χ^2 =^ 1.047, p = 0.593, df = 2).

**Table 3:** Baseline demographic and clinical characteristics for the three groups (mean ± SD/n,%).

Characteristic		Group (n=98)			*F*/χ^2^	*p*
		Control (n=33)	RT (n=31)	BFRE (n=34)		
Age, years		65.55 ± 4.41	66.65 ± 4.94	66.41 ± 4.97	0.477	0.622
Height, m		162.12 ± 9.40	161.06 ± 7.92	161.38 ± 7.67	0.136	0.873
Weight, kg		70.06 ± 9.61	67.81 ± 9.70	68.79 ± 9.42	0.447	0.641
Sex	Male	14(42.4)	13(41.9)	12(35.3)	0.442	0.802
	Female	19(57.6)	18(58.1)	22(64.7)		
Medications used for dyslipidemia	Yes	11(33.3)	8(25.8)	13(38.2)	1.150	0.563
	No	22(66.7)	23(74.2)	21(61.8)		
Medications used for blood pressure	Yes	25(75.8)	21(67.7)	23(67.6)	0.683	0.711
	No	8(24.2)	10(32.3)	11(32.4)		
Course of diabetes, years	2–5	11(33.3)	13(41.9)	16(47.0)	5.896	0.207
	5–10	18(54.5)	12(38.7)	9(26.5)		
	>10	4(12.2)	6(19.4)	9(26.5)		
Glucose-lowering medication	Oral medication	22(66.7)	16(51.6)	23(67.6)	2.506	0.644
	Oral medication + insulin	7(21.2)	11(35.5)	8(23.6)		
	Other	4(12.1)	4(12.9)	3(8.8)		
Blood pressure, mmHg	Systolic	127.73 ± 10.41	128.68 ± 14.07	126.65 ± 14.38	0.196	0.822
	Diastolic	79.97 ± 7.02	78.13 ± 7.61	79.12 ± 8.19	0.446	0.629
Blood lipid components, mmol/L	TC	4.70 ± 0.87	4.54 ± 1.08	4.63 ± 0.97	0.225	0.799
	TG	2.60 ± 0.74	2.51 ± 0.82	2.46 ± 0.89	0.247	0.782
	HDL-C	1.20 ± 0.29	1.12 ± 0.32	1.21 ± 0.38	0.710	0.494
	LDL-C	2.92 ± 0.63	2.85 ± 0.52	2.74 ± 0.61	0.424	0.655
Blood glucose indicators	FPG, mmol/L	8.55 ± 1.12	8.14 ± 1.42	8.36 ± 1.46	0.761	0.470
	HbA1c,%	7.98 ± 0.96	7.80 ± 0.93	7.75 ± 0.97	0.544	0.582
Body composition	WHtR, %	0.56 ± 0.03	0.56 ± 0.04	0.55 ± 0.03	0.727	0.486
	BMI, kg/m^2^	26.56 ± 1.58	26.02 ± 2.05	26.31 ± 1.88	0.675	0.512
	FM, kg	24.79 ± 3.42	22.97 ± 4.42	23.72 ± 4.60	1.540	0.220
	FMI, kg/m^2^	9.49 ± 1.49	8.83 ± 1.48	9.08 ± 1.42	1.644	0.199
	Lean mass, kg	45.27 ± 8.57	44.84 ± 6.37	45.07 ± 6.70	0.028	0.972
	SMI, kg/m^2^	8.79 ± 0.80	8.91 ± 0.71	8.96 ± 0.71	0.436	0.648
	ASMI, kg/m^2^	6.39 ± 0.60	6.47 ± 0.55	6.54 ± 0.54	0.559	0.574
Muscle performance	Handgrip strength, kg	23.08 ± 5.18	22.94 ± 4.37	24.04 ± 5.20	0.484	0.618
	5 times sit-stand test, s	16.26 ± 3.09	15.72 ± 2.45	15.97 ± 2.47	0.323	0.725
	6-m walk test, m/s	1.08 ± 0.07	1.09 ± 0.07	1.07 ± 0.08	0.563	0.571

BFRE, blood flow-restrictive resistance exercise group; RT, moderate-intensity resistance training group; FPG, fasting plasma glucose; HbA1c, Glycosylated hemoglobin; TC, total cholesterol; TG, triglycerides; HDL-C, high-density lipoprotein cholesterol; LDLC, low-density lipoprotein cholesterol; WHtR, waist-to-height ratio; BMI, body mass index; SMM, skeletal muscle mass; ASM, appendicular skeletal muscle mass; FM, fat mass; LM, lean mass; FMI, fat mass index; SMI, skeletal muscle mass index; ASMI, appendicular skeletal muscle mass index.

### 3.3 Results of repeated measures analysis of variance

**Tables 4**–**7** report the results of two-factor repeated measures of ANOVA used to compare and evaluate differences in FPG, HbA1C, BP, lipid profile, body composition, and muscle performance of the three groups. According to the Shapiro-Wilk test, all dependent variables basically obeyed normal distribution (p > 0.05). According to the Mauchly’s sphericity test, except for HbA1c, DBP, LDL-C, and ASMI, all other dependent variables did not meet the sphericity hypothesis (p< 0.05). The dependent variables that did not meet the sphericity hypothesis were subject to the results of Greenhouse-Geisser correction. The Levene test showed that the variance of the dependent variables was homogeneous (p > 0.05). The results of repeated measures ANOVA showed that except for the (group × time) interaction effect and the main effects of group and time on lean mass, which were not significant (p > 0.05), as well as the main effect of group on SBP, which was not significant (p > 0.05), the remaining dependent variables were significantly affected by the (group × time) interaction effect, and the main effects of group and time (p< 0.05).

**Table 4:** Effects of interventions on FPG, HbA1c, and BP among the three groups (mean ± SD or mean difference [95% CI]).

Index and time points	Group (n=98)			Cohen’s d			Repeated measurement analysis of variance			
	Control (n=33)	RT (n=31)	BFRE (n=34)	RT versus Control	BFRE versus Control	RT versus BFRE		Group × Time Interaction	Time	Group
Fasting plasma glucose (FPG), mmol/L										
T0	8.55 ± 1.12	8.14 ± 1.42	8.36 ± 1.46	−0.32	−0.15	−0.15	*F*	5.205	37.529	3.205
T3	8.48 ± 1.12	7.70 ± 1.38	7.82 ± 1.08	−0.62*	−0.6	−0.10	p	< 0.001	< 0.001	0.045
T6	8.41 ± 1.20	7.51 ± 1.13	7.63 ± 0.90	−0.77*	−0.74*	−0.12	η^2^P	0.099	0.283	0.063
T3 versus T0	−0.07(−0.29,0.15)	−0.44(−0.67,−0.21)#	−0.54(−0.76,−0.32)#							
T6 versus T0	−0.15(−0.43,0.14)	−0.62(−0.91,−0.33)#	−0.73(−1.01,−0.46)#							
Glycosylated hemoglobin (HbA1c), %										
T0	7.98 ± 0.96	7.80 ± 0.93	7.75 ± 0.97	−0.19	−0.24	0.05	*F*	20.839	96.393	3.484
T3	7.95 ± 0.92	7.37 ± 0.85	7.53 ± 0.93	−0.65*	−0.45	−0.18	*p*	< 0.001	< 0.001	0.035
T6	7.93 ± 0.75	7.11 ± 0.75	7.24 ± 0.85	−1.09#	−0.86*	−0.16	η^2^P	0.305	0.504	0.068
T3 versus T0	−0.04(−0.17,0.09)	−0.43(−0.56,−0.30)#	−0.22(−0.34,−0.10)#							
T6 versus T0	−0.05(−0.18,0.08)	−0.69(−0.83,−0.55)#	−0.51(−0.64,−0.38)#							
Systolic blood pressure (SBP), mmHg										
T0	127.73 ± 10.41	128.68 ± 14.07	126.65 ± 14.38	0.08	−0.09	0.14	*F*	4.589	9.897	0.915
T3	128.70 ± 13.38	125.19 ± 12.40	124.09 ± 12.43	−0.27	−0.36	0.09	*p*	0.003	< 0.001	0.404
T6	128.39 ± 10.21	124.71 ± 12.35	122.29 ± 10.85	−0.33	−0.58	0.21	η^2^P	0.088	0.094	0.019
T3 versus T0	0.97(−1.55, 3.49)	−3.48(−6.08,−0.89)*	−2.56(−5.04,−0.08)*							
T6 versus T0	0.67(−2.16, 3.50)	−3.97(−6.89,−1.05)*	−4.35(−7.14,−1.56)*							
Diastolic blood pressure (DBP), mmHg										
T0	79.97 ± 7.02	78.13 ± 7.61	79.12 ± 8.19	−0.25	0.11	−0.13	*F*	8.130	8.368	3.263
T3	81.06 ± 6.81	76.23 ± 7.57	76.97 ± 9.31	−0.67	−0.5	−0.09	*p*	< 0.001	< 0.001	0.043
T6	81.42 ± 5.93	75.19 ± 7.29	75.94 ± 7.50	−0.94*	−0.81*	−0.10	η^2^P	0.146	0.081	0.064
T3 versus T0	1.09(−0.61, 2.79)	−1.90(−3.66,−0.15)*	−2.15(−3.82,−0.47)*							
T6 versus T0	1.46(−2.22, 3.13)	−2.94(−4.66,−1.21)#	−3.18(−4.79,−1.56)#							

BFRE, blood flow-restrictive resistance exercise group; RT, moderate-intensity resistance training group; η^2^P, partial eta-squared; T0, baseline; T3, at third month; T6, at sixth month; *, significant difference at p< 0.05; #, significant difference at p< 0.001.

**Table 5:** Effects of interventions on blood lipids among the three groups [mean ± SD or mean difference (95% CI)].

Indexes and time points	Group (n=98)			Cohen’s d			Repeated measurement analysis of variance			
	Control (n=33)	RT (n=31)	BFRE (n=34)	RT versus Control	BFRE versus Control	RT versus BFRE		Group × Time Interaction	Time	Group
Total cholesterol (TC), mmol/L										
T0	4.70 ± 0.87	4.54 ± 1.08	4.63 ± 0.97	−0.16	−0.08	−0.09	*F*	4.266	7.241	4.608
T3	4.86 ± 0.80	4.27 ± 0.77	4.25 ± 0.79	−0.75*	−0.77*	0.03	*p*	0.005	0.002	0.012
T6	4.81 ± 0.81	4.14 ± 0.67	4.09 ± 0.63	−0.90#	−0.99#	0.08	η^2^P	0.082	0.071	0.088
T3 versus T0	0.16(−0.18, 0.50)	−0.26(−0.61, 0.09)	−0.38(−0.72, −0.50)*							
T6 versus T0	0.11(−0.23, 0.45)	−0.40(−0.74, −0.05)*	−0.54(−0.87, −0.20)#							
Triglyceride (TG), mmol/L										
T0	2.60 ± 0.74	2.51 ± 0.82	2.46 ± 0.89	−0.12	−0.17	0.06	*F*	5.578	13.236	3.691
T3	2.63 ± 0.77	2.27 ± 0.81	2.08 ± 0.70	−0.46	−0.75*	0.25	*p*	0.001	< 0.001	0.029
T6	2.67 ± 0.65	2.14 ± 0.78	2.00 ± 0.67	−0.74*	−1.02#	0.19	η^2^P	0.105	0.122	0.072
T3 versus T0	0.03(−0.23, 0.29)	−0.24(−0.51, 0.27)	−0.38(−0.63, −0.12)*							
T6 versus T0	0.07(−0.16, 0.30)	−0.37(−0.61, −0.14)#	−0.46(−0.69, −0.24)#							
High density lipoprotein cholesterol (HDL-C), mmol/L										
T0	1.20 ± 0.29	1.12 ± 0.32	1.21 ± 0.38	−0.26	0.03	−0.26	*F*	10.219	64.361	6.777
T3	1.23 ± 0.31	1.35 ± 0.32	1.52 ± 0.33	0.38	0.91*	−0.52	*p*	< 0.001	< 0.001	0.002
T6	1.27 ± 0.31	1.58 ± 0.30	1.70 ± 0.33	1.02#	1.34#	−0.38	η^2^P	0.177	0.404	0.125
T3 versus T0	0.04(−0.10, 0.18)	0.23(0.09, 0.38)#	0.31(0.17, 0.45)#							
T6 versus T0	0.08(0.05, 0.20)	0.46(0.33, 0.59)#	0.50(0.37, 0.62)#							
Low density lipoprotein cholesterol (LDL-C), mmol/L										
T0	2.92 ± 0.63	2.85 ± 0.52	2.78 ± 0.61	−0.12	−0.23	0.12	*F*	4.834	21.146	3.544
T3	2.90 ± 0.86	2.49 ± 0.62	2.51 ± 0.59	−0.54	−0.53	−0.03	*p*	0.001	< 0.001	0.033
T6	2.90 ± 0.92	2.41 ± 0.51	2.35 ± 0.53	−0.65*	−0.74*	0.12	η^2^P	0.092	0.182	0.069
T3 versus T0	−0.02(−0.20,0.17)	−0.36(−0.55, −0.18)#	−0.27(−0.46, 0.09)*							
T6 versus T0	−0.02(−0.23,0.21)	−0.45(−0.66, −0.23)#	−0.43(−0.64, −0.23)#							

BFRE, blood flow-restrictive resistance exercise group; RT, moderate-intensity resistance training group; η^2^P, partial eta-squared; T0, baseline; T3, at third month; T6, at sixth month; *, significant difference at p<0.05; #, significant difference at p< 0.001.

**Table 6:** Effects of interventions on body composition among the three groups [mean ± SD or mean difference (95% CI)].

Indexs and time points	Group (n=98)					Cohen’s d			Repeated measurement analysis of variance			
	Control (n=33)	RT (n=31)			BFRE (n=34)	RT versus Control	BFRE versus Control	RT versus BFRE		Group × Time Interaction	Time	Group
Waist-to-height ratio (WHtR), kg/m												
T0	0.56 ± 0.03		0.56 ± 0.04	0.55 ± 0.03		0.00	−0.33	0.29	*F*	143.912	240.453	8.817
T3	0.56 ± 0.03		0.54 ± 0.04	0.53 ± 0.03		−0.57*	−1.00#	0.29	*p*	<0.001	<0.001	<0.001
T6	0.57 ± 0.03		0.51 ± 0.03	0.51 ± 0.03		−1.67#	−1.67#	0.00	η^2^P	0.752	0.717	0.157
T3 versus T0	0.003(−0.001, 0.007)		−0.02(−0.03, −0.02)#	−0.03(−0.03, −0.02`)#								
T6 versus T0	0.01(0.01, 0.02)#		−0.05(−0.05, −0.04)#	−0.04(−0.04, −0.03)#								
Body mass index (BMI), kg/m^2^												
T0	26.56 ± 1.58		26.02 ± 2.05	26.31 ± 1.88		−0.30	−0.14	−0.15	*F*	129.249	205.911	8.198
T3	27.00 ± 1.53		25.22 ± 1.98	25.73 ± 1.92		−1.01#	−0.73*	−0.26	*p*	< 0.001	< 0.001	< 0.001
T6	27.11 ± 1.61		24.14 ± 1.95	24.82 ± 1.88		−1.67#	−1.31#	−0.36	η^2^P	0.731`	0.684	0.147
T3 versus T0	0.44(0.30, 0.59)#		−0.80(−0.96, −0.65)#	−0.58(−0.72, −0.43)#								
T6 versus T0	0.56(0.31, 0.80)#		−1.88(−2.13, −1.63)#	−1.48(−1.72, −1.24)#								
Fat mass (FM), kg												
T0	24.79 ± 3.42		22.97 ± 4.42	23.72 ± 4.60		−0.46	−0.26	−0.17	*F*	99.645	130.536	20.502
T3	26.15 ± 3.12		20.41 ± 4.07	21.87 ± 4.37		−1.59#	−1.12#	−0.35	*p*	<0.001	<0.001	<0.001
T6	26.80 ± 3.19		17.38 ± 3.59	19.25 ± 3.42		−2.78#	−2.28#	−0.53	η^2^P	0.677	0.579	0.301
T3 versus T0	1.36(0.68, 2.04)#		−2.56(−3.26, −1.85)#	−1.87(−2.51, −1.22)#								
T6 versus T0	2.01(1.11, 2.90)#		−5.59(−6.51, −4.67)#	−4.47(−5.28, −3.66)#								
Lean mass (LM), kg												
T0	45.27 ± 8.57		44.84 ± 6.37	45.07 ± 6.70		−0.06	−0.03	−0.04	*F*	2.361	1.065	0.023
T3	45.06 ± 8.14		45.30 ± 6.59	45.44 ± 6.72		0.03	0.05	−0.02	*p*	0.062	0.341	0.977
T6	44.72 ± 8.44		45.56 ± 6.88	45.69 ± 7.14		0.11	0.12	−0.02	η^2^P	0.047	0.011	0.000
T3 versus T0	−0.21(−0.95,0.53)		0.46(−0.30, 1.22)	0.37(−0.34, 1.07)								
T6 versus T0	−0.55(−1.57,0.46)		0.72(−0.33, 1.77)	0.62(−0.28, 1.52)								
Fat mass index (FMI), kg/m^2^												
T0	9.49 ± 1.49		8.83 ± 1.48	9.08 ± 1.42		−0.44	−0.28	−0.17	*F*	106.561	143.034	24.246
T3	9.98 ± 1.19		7.86 ± 1.42	8.36 ± 1.32		−1.62#	−1.29#	−0.37	*p*	<0.001	<0.001	<0.001
T6	10.23 ± 1.31		6.68 ± 1.21	7.37 ± 1.06		−2.81#	−2.40#	−0.61	η^2^P	0.692	0.601	0.338
T3 versus T0	0.49(0.24, 0.74)#		−0.98(−1.24, −0.72)#	−0.72(−0.95, −0.48)#								
T6 versus T0	0.75(0.43, 1.07)#		−2.15(−2.49, −1.82)#	−1.70(−2.00, −1.41)#								
Skeletal muscle mass index (SMI), kg/m^2^												
T0	8.79 ± 0.80		8.91 ± 0.71	8.96 ± 0.71		0.16	0.23	−0.07	*F*	11.825	19.644	3.910
T3	8.71 ± 0.87		9.19 ± 0.58	9.23 ± 0.84		0.65*	0.61*	−0.06	*p*	<0.001	<0.001	0.023
T6	8.66 ± 0.93		9.41 ± 0.71	9.34 ± 0.85		0.90*	0.76*	0.09	η^2^P	0.199	0.171	0.076
T3 versus T0	−0.08(−0.25,0.09)		0.28(0.11, 0.45)#	0.27(0.12, 0.43)#								
T6 versus T0	−0.13(−0.34,0.09)		0.50(0.28, 0.72)#	0.38(0.19, 0.57)#								
Appendicular skeletal muscle mass index (ASMI), kg/m^2^												
T0	6.39 ± 0.60		6.47 ± 0.55	6.54 ± 0.54		0.14	0.26	−0.13	*F*	28.648	47.298	6.411
T3	6.30 ± 0.65		6.71 ± 0.53	6.82 ± 0.64		0.69*	0.81*	−0.19	*p*	<0.001	<0.001	0.002
T6	6.24 ± 0.70		6.99 ± 0.54	7.00 ± 0.65		1.20#	1.13#	−0.02	η^2^P	0.376	0.332	0.119
T3 versus T0	−0.09(−0.21,0.03)		0.24(0.17, 0.36)#	0.29(0.18, 0.39)#								
T6 versus T0	−0.15(−0.31,0.01)		0.52(0.36, 0.67)#	0.46(0.33, 0.59)#								

BFRE, blood flow-restrictive resistance exercise group; RT, moderate-intensity resistance training group; η^2^P, partial eta-squared; T0, baseline; T3, at third month; T6, at sixth month; *, significant difference at p< 0.05; #, significant difference at p< 0.001.

**Table 7:** Effects of interventions on muscle performance among three groups [mean ± SD or mean difference (95% CI)].

Indexs and time points	Group (n=98)			Cohen’s d			Repeated measurement analysis of variance			
	Control (n=33)	RT (n=31)	BFRE (n=34)	RT versus Control	BFRE versus Control	RT versus BFRE		Group × Time Interaction	Time	Group
Grip strength test, kg										
T0	23.08 ± 5.18	22.94 ± 4.37	24.04 ± 5.20	−0.03	0.19	−0.23	*F*	240.830	601.558	3.149
T3	22.78 ± 4.88	26.06 ± 4.33	25.58 ± 5.00	0.71*	0.57	0.10	*p*	<0.001	<0.001	0.047
T6	22.70 ± 4.77	27.41 ± 4.37	26.68 ± 5.32	1.03#	0.79*	0.15	η^2^P	0.835	0.864	0.062
T3 versus T0	−0.29(−0.57,−0.02)#	3.12(2.84, 3.40)#	1.54(1.27, 1.81)#							
T6 versus T0	−0.38(−0.69,−0.06)#	4.47(4.15, 4.80)#	2.64(2.34, 2.96)#							
Five times-sit-to-stand test, s										
T0	16.26 ± 3.09	15.72 ± 2.45	15.97 ± 2.47	−0.19	−0.10	−0.10	*F*	49.948	103.534	3.833
T3	16.48 ± 3.22	14.60 ± 2.14	14.84 ± 2.38	−0.68*	−0.58*	0.11	*p*	<0.001	<0.001	0.025
T6	16.61 ± 3.02	14.26 ± 2.23	14.13 ± 2.22	−0.88#	−0.94#	0.06	η^2^P	0.513	0.521	0.075
T3 versus T0	0.22(−0.09, 0.54)	−1.11(−1.44,−0.79)#	−1.13(−1.44,−0.82)#							
T6 versus T0	0.35(0.05, 0.67)#	−1.45(−1.78,−1.14)#	−1.85(−2.15,−1.54)#							
6-m walking speed, s/m										
T0	1.08 ± 0.07	1.09 ± 0.07	1.07 ± 0.08	0.14	−0.13	0.27	*F*	40.884	99.069	5.087
T3	1.08 ± 0.08	1.13 ± 0.06	1.11 ± 0.05	0.70*	0.45	0.36	*p*	<0.001	<0.001	0.008
T6	1.07 ± 0.07	1.16 ± 0.07	1.15 ± 0.04	1.29#	1.41#	0.18	η^2^P	0.463	0.510	0.097
T3 versus T0	−0.01(−0.02,0.01)	0.04(0.03, 0.06)#	0.05(0.03, 0.06)#							
T6 versus T0	−0.01 (−0.03, 0.003)	0.07(0.06, 0.09)#	0.08(0.07, 0.10)#							

BFRE, blood flow-restrictive resistance exercise group; RT, moderate-intensity resistance training group; η^2^P, partial eta-squared; T0, baseline; T3, at third month; T6, at sixth month; *, significant difference at p< 0.05; #, significant difference at p< 0.001.

### 3.4 Primary outcomes

**Table 4** shows the changes in FPG, HbA1c, and BP for the three groups. The results of repeated measures ANOVA indicate that the main effect of time in FPG, HbA1c, SBP and DBP were significant (p< 0.001). Compared to baseline, from the third month of intervention, the FPG, HbA1c, SBP and DBP of the two exercise groups decreased significantly from baseline (p< 0.05), and the improvement effect became more significant over time. Compared to the control group, the FPG (d = -0.62, p = 0.032) and HbA1c (d = -0.65, p = 0.038) in the RT group began to significantly decrease at the third month of intervention, while the same effect occurred in the BFRE group at the sixth month of intervention. At the sixth month of intervention, there was no significant difference in SBP between the three groups (F = 0.915, p = 0.404, η^2^P = 0.019), while the FPG, HbA1c, and DBP in RT and BFRE were significantly decreased compared to the control group (p< 0.05), with no significant difference between the two exercise groups (p > 0.05).

**Table 5** shows the changes in blood lipids. Compared with baseline, at the third month of intervention, TC [with a mean (95% CI) change of -0.38 (-0.72, -0.50), p = 0.018] and TG [-0.38, (-0.63, -0.12), p = 0.002] in the BFRE group decreased significantly, while HDL-C increased significantly and LDL-C decreased significantly in both exercise groups (p< 0.05). At the sixth month of intervention, TC, TG, and LDL-C decreased significantly and HDL-C increased significantly in the two exercise groups (p< 0.05), while the improvement of blood lipids in the control group were not significant (p > 0.05). Compared with the control group, at the third month of intervention, TC in both exercise groups decreased significantly [(RT, d = -0.75, p = 0.011), (BFRE, d = -0.77, p = 0.006)], and TG decreased (d = -0.75, p = 0.012) and HDL-C increased (d = 0.91, p = 0.002) significantly in the BFRE group. At the sixth month of intervention, dyslipidemia in both exercise groups improved significantly compared with the control group (p< 0.05), with no significant difference in blood lipids between the two exercise groups (p > 0.05).

**Table 6** shows the changes in body composition. The results of repeated measures ANOVA indicate that the main effect of time and the interaction effect of (group × time) on WHtR, BMI, FM, FMI, SMI, ASMI were significant (p< 0.001), indicating that the improvement effect of exercise intervention on these indicators became more pronounced over time. Compared with baseline, from the third month of intervention, WHtR, BMI, FM, FMI, SMI, ASMI showed significant improvements in both exercise groups (p< 0.05). At the third month of intervention, increases in SMI [RT, 0.28 (0.11, 0.45); BFRE, 0.27 (0.12, 0.43)] and ASMI [RT, 0.24 (0.17, 0.36); BFRE, 0.29 (0.18, 0.39)] in both exercise groups was similar, while at the sixth month of intervention, the SMI and ASMI increase in the BFRE group [SMI, 0.38 (0.19, 0.57); ASMI, 0.46 (0.33, 0.59)] was lower than that of the RT group [SMI, 0.50 (0.28, 0.72), ASMI, 0.52 (0.36, 0.67)]. At the sixth month follow-up, WHtR, BMI, FM, FMI in the control group were significantly higher than baseline (p< 0.05). Compared with the control group, WHtR, BMI, FM, FMI, SMI, ASMI in two exercise groups were significantly improved since third month of intervention (p< 0.05). There were no significant differences in body composition between the two exercise groups (p > 0.05).

### 3.5 Secondary outcomes

**Table 7** shows the muscle performance data. Compared with the baseline, the muscle performance of both exercise groups significantly increased from the third month of intervention (p< 0.05), with the grip strength of the RT group increasing by 1.7–2 times that of the BFRE group. At the sixth month of follow-up, the performance of the control group in the grip strength and five times-sit-to-stand tests significantly decreased than baseline (p< 0.05). Compared with the control group, the two exercise groups showed significantly better performance in five times-sit-to-stand test at the third month of intervention (p< 0.05). At the same time, the grip strength (d = 0.71, p = 0.021) and 6-m walking speed (d = 0.70, p = 0.005) of the RT group significantly increased compared with the control group, while the same improvement effect was observed in the BFRE group at the sixth month of intervention. There was no significant difference in muscle performance between the two exercise groups (p > 0.05).

### 3.6 Safety outcomes

Adverse events in this study were defined as any adverse symptoms or events related to the study measures or exercise intervention that occurred during the study. Serious adverse events were defined as those that were life-threatening, fatal, or resulted in permanent disability. During the research period, four events were defined as adverse events, with similar incidence rates in each group (RT, n = 2; BFRE, n = 1; control group, n = 1). One person in the RT group developed lateral epicondylitis of the humerus, which may be related to inappropriate force during resistance training and housework. After rest and treatment, her symptoms had been resolved. One participant in each of the RT and BFRE groups experienced similar symptoms of hypoglycemia at home, but their self-measured blood glucose levels were 4.1 mmol/L and 4.3 mmol/L, respectively, at the time. The symptoms resolved after eating, which was considered to be related to increased physical activity but not timely eating. One person in the control group experienced dizziness during a blood test taken while fasting, which may be due to excessive tension. In addition, the safety testing and two-week of adaptation training were completed before the exercise intervention. Those who could not continue due to illness or other reasons withdrew. No abnormal fluctuations in blood pressure or blood glucose occurred during exercise, and no serious adverse events, such as syncope, thrombosis, subcutaneous bleeding, falls, cardiovascular and cerebrovascular accidents occurred.

## 4 Discussion

The preliminary finding of this randomized controlled study was that for older adults with T2DM, BFRE was not only safe and easy to implement, but also achieved similar effects to moderate-intensity resistance training in improving patients’ FPG, HbA1c, dyslipidemia, blood pressure, body composition, and muscle performance. Although there was not significant difference between the BFRE and RT in improving metabolic disorder and body composition in older adults with T2DM, from the trend of data changes, The BFRE had a faster effect in improving dyslipidemia, and the magnitude of increase in muscle mass and strength of the RT was greater.

Our results showed that moderate-intensity resistance exercise could significantly improve the FPG and HbA1c in older adults with T2DM. This was consistent with many previous research findings (26, 27). However, it is worth noting that the dropout rate of the RT group reached 34.04% in this study, and the main reason for withdrawal was feeling fatigued and sore in the early stages of exercise, which made them worried about worsening joint and muscle damage. In the cognition of older adults, resistance training is more suitable for those who are muscular, and participation in resistance training may increases the risk of having a heart attack, stroke or death in the elderly (6). Although the likelihood of these occurrences is small, it hinders their participation in resistance training (28).

Our research designed a BFRE program for older adults with T2DM. The main purpose of this design was to lower the load of resistance training, reduce the initial intensity and fatigue, and quickly obtain the protective effect of resistance training on blood glucose control. The results of this study show that six months of BFRE could effectively improve the FPG and HbA1c in older adults with T2DM. The effect was similar to that of moderate-intensity resistance exercise. Previous studies (14) have confirmed that BFRE can restrict blood flow by continuously pressurizing the limbs, causing severe fluctuations in the redox state of muscles due to ischemia and hypoxia, and increasing the high accumulation of reactive oxygen species (ROS). ROS activates 5’-AMP-activated protein kinase, enhancing GLUT4 mRNA and protein expression, increasing the abundance of GLUT4 in human skeletal muscle, and thus increasing glucose uptake. Therefore, most studies on BFRE use continuous pressure on the limbs during training to stimulate skeletal muscle hypertrophy and increase glucose uptake, but this can significantly increase exercise fatigue in subjects (13, 29, 30). Wang et al. (31) found that continuous compression during BFRE can improve muscle functional capacity more than intermittent compression, but higher fatigue phenomena also occur. In contrast, although intermittent BFRE can also bring fatigue, the recovery speed is faster. It is recommended that beginners adopt intermittent BFRE. Husmann et al. (32) pointed out that BFRE exacerbated the accumulation of exercise-induced, fatigue-related metabolites and prevented the recovery of contractile function during rest intervals. However, after two minutes of reperfusion, muscle contraction function recovered substantially, diminishing the impact of blood flow restriction on muscle fatigue. Therefore, in our BFRE program, there were 6.5 minutes of continuous compression on both the upper or lower limbs to stimulate skeletal muscle hypertrophy and increase skeletal muscle glucose uptake. Additionally, there were two minutes of blood flow reperfusion to reduce fatigue, allowing subjects to complete longer single-training sessions (30 minutes), fully exercise the muscles and joints of the body, and enhance exercise endurance.

In our study, although there was no significant difference in SBP between the two exercise groups and the control group, there was a significant decrease in both systolic and diastolic blood pressure compared to baseline. This shows that BFRE can steadily reduce the blood pressure of the older adults with T2DM. The impact of BFRE on blood pressure is still controversial. The experimental results of Rossow et al. (33) showed that only high-intensity resistance exercise showed a good antihypertensive effect on young men, while the effect of BFRE was not significant. The research results of Crisafulli et al. (34) and Maior et al. (35) indicate that young men only need to use relatively small amounts of muscles (such as grasping, biceps curling, or single-joint exercise) for low-load BFRE (≤ 40% 1-RM) to reduce post-exercise blood pressure. The meta-analysis results of Domingos et al. (36) suggested that although BFRE lead to greater post-exercise hypotension compared to traditional exercise, higher SBP and/or DBP were observed during BFRE, especially in hypertensive patients. Therefore, caution should be exercised when using BFRE. Another study suggests (37) that for older adults, the acute hemodynamic response caused by low-intensity BFRE is similar to that caused by high-load training, and can return to normal levels within 30 minutes after training with a more significant decrease in SBP. However, these studies were aimed at young adults and the non-diabetes population. The effect of BFRE on blood pressure in older adults with T2DM is rarely reported. Given previous studies suggesting that BFRE may cause fluctuations in blood pressure during exercise, in this study, we monitored the blood pressure of participants before exercise and found that abnormally elevated blood pressure may be related to factors such as climate change, poor sleep, and underlying disease changes. We tracked and treated patients with high blood pressure as necessary, so there were no significant fluctuations in blood pressure during exercise. After the six-month intervention, the blood pressure of participants in the BFRE group slowly declined with the extension of exercise time, but it did not cause a sudden drop in blood pressure, which has a protective effect on the cardiovascular health of the elderly.

In our research results, after exercise intervention, there was no significant change in lean mass, but WHtR, BMI, FM, and FMI were significantly reduced, and dyslipidemia was significantly improved in the RT and BFRE groups. This indicated that the two types of exercise could effectively reduce fat, especially abdominal fat, to achieve the goal of optimizing body shape and blood lipids. This is consistent with the research findings of Sun et al. (38). Abdominal obesity in elderly T2DM patients is often accompanied by more serious dyslipidemia (39, 40). The increased LDL-C has a lower affinity for vascular endothelial tissue and arterial wall proteoglycans, and is more prone to oxidation, leading to the formation of atherosclerotic plaques in the arteries (41). HDL-C dysfunction leads to the reduction of its anti-atherosclerosis, antioxidant, and anti-inflammatory properties, and accelerates the process of atherosclerosis (42). Elderly T2DM patients should be encouraged to exercise regularly in various ways to change abdominal obesity and dyslipidemia, in order to reduce the risk of cardiovascular complications. Compared to moderate-intensity resistance training, BFRE has a lower load during training, allowing athletes to use portable and lightweight exercise equipment to complete a variety of movements. This makes exercise more enjoyable and interesting without being limited by venue and time, making it more conducive to long-term persistence in elderly T2DM patients.

In this study, the SMI and ASMI of the two exercise groups were significantly improved compared to the baseline and control group, and the effects of the two exercise groups were similar. This may be because the hypoxic environment created by BFRE increases blood lactate concentration, accumulates metabolites, and promotes increased secretion of muscle hypertrophy hormones (such as growth hormone), synergistically promoting muscle protein synthesis (43). In addition, BFRE may further promote muscle hypertrophy by affecting the generation of nitric oxide or the activation of specific heat shock proteins (44). Therefore, even if low-intensity exercise were used, it could also achieve the muscle strengthening effect of moderate-intensity resistance training, which is of great significance to older adults with T2DM to prevent and treat sarcopenia. However, Lixandrão et al. (45) pointed out that muscle hypertrophy does not necessarily mean an increase in muscle strength. Even if both exercises can induce similar muscle mass increases, high-intensity resistance training (> 65% 1-RM) significantly improves muscle strength growth compared to low-intensity BFRE. This may be because low-intensity BFRE has a relatively low promoting effect on neuromuscular driving ability. The ability to recruit muscle fibers cannot achieve the same effect as high-intensity training. In our study, although the increase in grip strength in the RT group was 1.7–2 times that of the BFRE group, there was no significant difference in grip strength, sit-to-stand and walking speed between the two exercise groups. This may be because our research mainly focused on older adults with T2DM. The strength and speed improvement of elderly people with sedentary habits was relatively limited, and diabetes patients tend to be weaker and more prone to fatigue. BFRE puts skeletal muscles in a hypoxic environment, leading to premature fatigue of type I muscle fibers. To resist external stress, more type II muscle fibers are activated to participate in work, and type II muscle fibers are the key to muscle hypertrophy and muscle strength growth. BFRE can improve the muscle mass and strength of older adults with T2DM with less effort, which is essential to preventing falls during exercise. After older adults improve their physical strength, balance, and stability through BFRE to a certain extent, their acceptance of exercise will be greatly enhanced, laying a good foundation for further acceptance of moderate-to-high-intensity resistance exercise or the other exercises.

## 5 Limitations of the study

This study also has some limitations, such as the intake of protein, carbohydrates, fat, and water in food affecting changes in lean mass and muscle mass. However, we did not provide a detailed evaluation of the participants’ dietary behaviors or nutritional content of the food consumed, as recording was difficult for older adults. This makes it difficult to determine why six months of training did not significantly increase the lean mass of participants. A possible reason is that to control blood glucose, the participants mainly consumed light vegetables in their diet, with a reduced proportion of fruits, staple foods, meat, and fats. In addition, age-related muscle loss slowed down the growth of lean mass after exercise to a certain extent. In the future, the impact of combining dietary control with BFRE on T2DM will be explored further, in hopes of better promoting this exercise.

## 6 Conclusion

In this study, a BFRE program was designed combining intermittent restriction of limbs blood flow and low-intensity resistance training. The results showed that BFRE could effectively improve the metabolic disorder of blood glucose, dyslipidemia, and blood pressure in older adults with T2DM, and could also enhance the muscle function of patients by controlling abdominal obesity and reducing muscle loss. The effects of BFRE were similar to moderate-intensity resistance training. However, from the perspective of long-term data trends, BFRE may not be as effective as moderate-intensity resistance exercise in balancing muscle mass and strength in patients. Therefore, it is suggested that the older adults with T2DM should use BFRE for training under the guidance of professional medical staff at the beginning of exercise to obtain exercise adaptation in this relatively simple and easy way, then consider carrying out moderate- intensity resistance training, and choosing proper exercise methods according to their own conditions to better control diabetes.

## Data availability statement

The original contributions presented in the study are included in the article/supplementary material. Further inquiries can be directed to the corresponding authors.

## Ethics statement

This study protocol has been reviewed and approved for implementation by the Medical Ethics Committee of Shaoyang University and registered on the Chinese Clinical Trials Registry (Registration No.: ChiCTR2300074357). The studies were conducted in accordance with the local legislation and institutional requirements. The participants provided their written informed consent to participate in this study. Written informed consent was obtained from the individual(s) for the publication of any potentially identifiable images or data included in this article.

## Author contributions

XM: Writing – review & editing, Writing – original draft, Visualization, Validation, Supervision, Software, Resources, Project administration, Methodology, Investigation, Funding acquisition, Formal analysis, Data curation, Conceptualization. YA: Writing – review & editing, Writing – original draft, Methodology. FL: Writing – review & editing, Writing – original draft, Investigation. XT: Writing – review & editing, Writing – original draft, Investigation. QL: Writing – review & editing, Writing – original draft, Investigation. YH: Writing – review & editing, Writing – original draft, Investigation. YZ: Writing – review & editing, Writing – original draft, Investigation. QM: Writing – review & editing, Writing – original draft, Investigation. LW: Writing – review & editing, Writing – original draft, Supervision, Methodology. FL: Writing – review & editing, Writing – original draft, Supervision, Methodology. QY: Writing – review & editing, Writing – original draft, Supervision, Methodology. FY: Writing – review & editing, Writing – original draft, Data curation. XY: Writing – review & editing, Writing – original draft, Software, Data curation. BH: Writing – review & editing, Writing – original draft, Supervision, Resources, Funding acquisition. LZ: Writing – review & editing, Writing – original draft, Software, Data curation. SR: Writing – review & editing, Writing – original draft, Supervision, Resources, Funding acquisition.
